# Factors Associated With Bites to a Child From a Dog Living in the Same Home: A Bi-National Comparison

**DOI:** 10.3389/fvets.2018.00066

**Published:** 2018-05-04

**Authors:** Locksley L. McV. Messam, Philip H. Kass, Bruno B. Chomel, Lynette A. Hart

**Affiliations:** ^1^Section: Herd Health and Animal Husbandry, School of Veterinary Medicine, College of Health and Agricultural Sciences, University College Dublin, Dublin, Ireland; ^2^Department of Population Health and Reproduction, School of Veterinary Medicine, University of California Davis, Davis, CA, United States

**Keywords:** dog bite, child, home, risk factor, cohort study, anthrozoology, human–animal interaction

## Abstract

We conducted a veterinary clinic-based retrospective cohort study aimed at identifying child-, dog-, and home-environment factors associated with dog bites to children aged 5–15 years old living in the same home as a dog in Kingston, Jamaica (236) and San Francisco, USA (61). Secondarily, we wished to compare these factors to risk factors for dog bites to the general public. Participant information was collected *via* interviewer-administered questionnaire using proxy respondents. Data were analyzed using log-binomial regression to estimate relative risks and associated 95% confidence intervals (CIs) for each exposure–dog bite relationship. Exploiting the correspondence between X% confidence intervals and X% Bayesian probability intervals obtained using a uniform prior distribution, for each exposure, we calculated probabilities of the true (population) RRs ≥ 1.25 or ≤0.8, for positive or negative associations, respectively. Boys and younger children were at higher risk for bites, than girls and older children, respectively. Dogs living in a home with no yard space were at an elevated risk (RR = 2.97; 95% CI: 1.06–8.33) of biting a child living in the same home, compared to dogs that had yard space. Dogs routinely allowed inside for some portion of the day (RR = 3.00; 95% CI: 0.94–9.62) and dogs routinely allowed to sleep in a family member’s bedroom (RR = 2.82; 95% CI: 1.17–6.81) were also more likely to bite a child living in the home than those that were not. In San Francisco, but less so in Kingston, bites were inversely associated with the number of children in the home. While in Kingston, but not in San Francisco, smaller breeds and dogs obtained for companionship were at higher risk for biting than larger breeds and dogs obtained for protection, respectively. Overall, for most exposures, the observed associations were consistent with population RRs of practical importance (i.e., RRs ≥ 1.25 or ≤0.8). Finally, we found substantial consistency between risk factors for bites to children and previously reported risk factors for general bites.

## Introduction

Children, particularly those younger than 10 years old, are generally considered to be at highest risk for dog bites. The immediate consequences of such events include both physical and mental trauma as well as infection by zoonotic agents (1–6). Studies have reported that children are more likely to be bitten in the face, neck, or head than adults, sometimes resulting in permanent scars and/or loss of function to sensitive areas of the body (2, 4–6). Posttraumatic stress disorder is also a potential sequel to a bite event, with some child-victims requiring psychological treatment and displaying emotional distress for extended periods (3, 4, 7). Quite likely because of their relatively small size, children are also over-represented among persons who are hospitalized or die consequent to a dog attack (2, 4–6, 8–11). A dog bite also threatens the welfare of the offending animal, as consequences often include removal from the home due to relinquishment to a shelter (12).

Most dog bites to children seem to occur at home by the family’s own dog (6, 13–15). This is not surprising given that the home is where both child and dog spend most of their day and, consequently, the most likely place where children who have dogs would interact with one. It is likely that characteristics of the home determine the types of contact occurring between child and dog, and whether these lead to a bite. Factors such as the presence or otherwise of yard space, the number of hours per day the dog is confined, leashed, or allowed into the house, and where it sleeps are all likely to affect the frequency and nature of daily child–dog contact. Additionally, other human- and canine-related home-environmental factors such as the presence of other children, other dogs, and the ages of both child and dog might contribute to the frequency and quality of daily child–dog interactions.

Given these observations, surprisingly, little research has focused on the home environment as a risk (or protective) factor for dog bite injuries, and no studies focusing on factors associated with dog bites to children in the context of the family home were found in the literature. From a prevention point of view, it is important to know to what extent home-environment characteristics are associated with family dog bites to the family child.

Previously, we reported on a retrospective cohort study comparing risk factors for general dog bites in Kingston, Jamaica, and San Francisco, USA (16–18). We now report on an investigation of a sub-cohort of 297 persons, from both cities, who resided in a household along with a child and dog. The aims of this particular analysis were threefold: first, to quantify associations between selected home-environment factors and the risk of a dog biting a child living in the same home; second, to evaluate the practical importance of these associations in the context of dog bites and third, to compare them to previously reported associations between these factors and dog bites in general (hereafter referred to as “general bites”). In maintaining the bi-national nature of the investigation, we also hoped to identify differences in risk (protective) factor—dog bite associations attributable to city of origin.

## Materials and Methods

### Study Protocol

This study was authorized by the University of California Davis’ Human Subjects Institutional Review Board and respondents provided verbal informed consent. Most aspects of the materials and methods are identical to those previously reported in detail (16–18). This report focuses on information gathered from a subset of persons (hereafter referred to as the respondents) who lived in a home with at least one child–dog pair (hereafter referred to as the participants).

Study respondents were clients interviewed in the waiting rooms of eight veterinary clinics in Kingston (KGN), Jamaica (May 30th - August 9th 2003), and three veterinary clinics in San Francisco (SF), USA (20th October 2003 - 10th January 2004) using identical questionnairesx (16). Respondents were required:

(a) To be 18 years or older,
(b) To have a dog present with them in the waiting room with which they lived 7 days a week, and
(c) To be living 7 days a week in the same home as a child aged 5–15 years of age for whom they were either a parent or guardian.

Whenever more than one dog was present, their names were ranked in alphabetical order and the dog with the first-ranked name was chosen. Similarly, when more than one child aged 5–15 years of age lived in the same home as the respondent, the children’s names were ranked alphabetically and the child with the first-ranked name was chosen for participation. This was done to reduce the possibility of selection bias resulting from preferential enrollment of either the dog- or child-participant based on the perceptions of the respondent. We restricted the age criterion to 5–15 years of age in order to render the child-participants’ age range as narrow as possible without limiting our ability to obtain a reasonably large sample. The presence of the child in the clinic was not a requirement for participation. If a respondent was accompanied by another person, that person was allowed to contribute to answering the interviewer’s questions, if the respondent wished. We chose to use proxy respondents rather than the index participants for several reasons; first, we wished to ensure that data obtained for younger children were of comparable quality to that obtained for older children. Second, study enrollment of minors (a vulnerable population) necessitates additional study participant-related safeguards that would have rendered data collection more time-consuming without any guaranteed increase in data quality. Third, in lieu of the index participant, this was the most efficient way to ensure that information was obtained from a person who could reliably report on both child and dog, as well as on the home environment. This was particularly advantageous, given that a substantial proportion of veterinary consultations occur while children are at school and unavailable.

### Outcome Determination

Dog bite categories were determined based on responses to the following questions:

(a) During play, in the last 2 years, did the dog ever hold onto or catch a part of the child in question’s body with its teeth and cause a wound?
(b) Not during play, in the last 2 years, did the dog ever hold onto or catch a part of the child in question’s body with its teeth and cause a wound?
(c) Not during play, in the last 2 years did the dog ever hold onto or catch a part of the child in question’s body with its teeth and not cause a wound?

The outcome was considered a bite if the respondent replied in the affirmative to one or more of a, b, or c, and a non-biter if the respondent replied in the negative to all three questions. When the respondent answered in the affirmative to more than one of the questions, the event that occurred earliest was chosen as the outcome. “During play” in this context referred to the behaviour of the child; no assumptions were made regarding whether or not the dog was playing. We assumed that respondents could accurately report on whether a child was playing with the dog but felt that this was not necessarily the case for when the dog was playing. We based this view on reports suggesting that dog-owners often misread the body language of dogs (19, 20).

### Exposure Information

Exposure information included characteristics of the respondent (e.g., age and sex), the 5 to 15-year-old child (e.g., age, sex, presence of disabilities) living in the same household as the dog, the child–dog interactions (e.g., whether the dog routinely avoided the child, frequency of energetic play, etc.), the dog (e.g., age, sex, and neuter status), and the child–dog home environment (e.g., number of children/dogs in home, presence of yard space, dog’s habitual sleeping location).

### Analysis

Data for 297 participants were used for final analyses in SPSS version 24. This included 22 bite victims with 13 children bitten during, and 9 bitten outside of play with the dog. In a previous report comparing bites occurring during and outside of play, we demonstrated that, from a point of view of the exposures examined, the two types of bites were not etiologically distinct (18). As the outcome and the majority of exposures used in this analysis were identical to those used in that report, bites that occurred “during play” and bites that occurred “not during play” were grouped together for analysis (hereafter referred to as “bites” or “child bites”).

**Figure 1:**
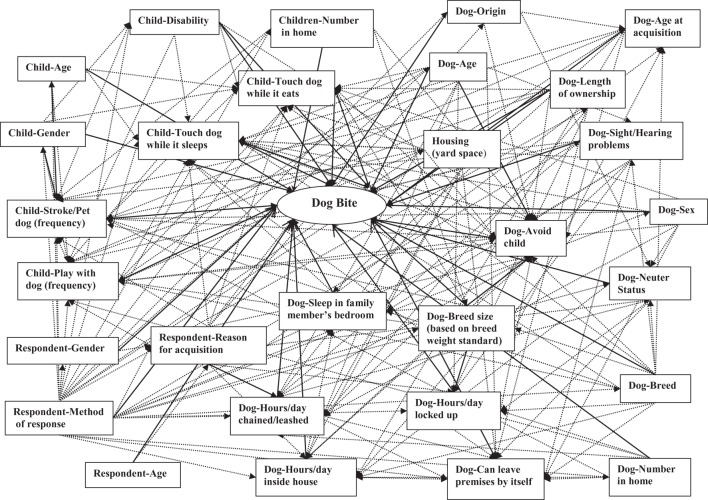
Master directed acyclic graph showing hypothesized causal web of dog bites. Solid lines represent hypothesized causal relationships between exposures and dog bites. Dotted lines represent hypothesized causal relationships between exposures.

**Figure 2:**
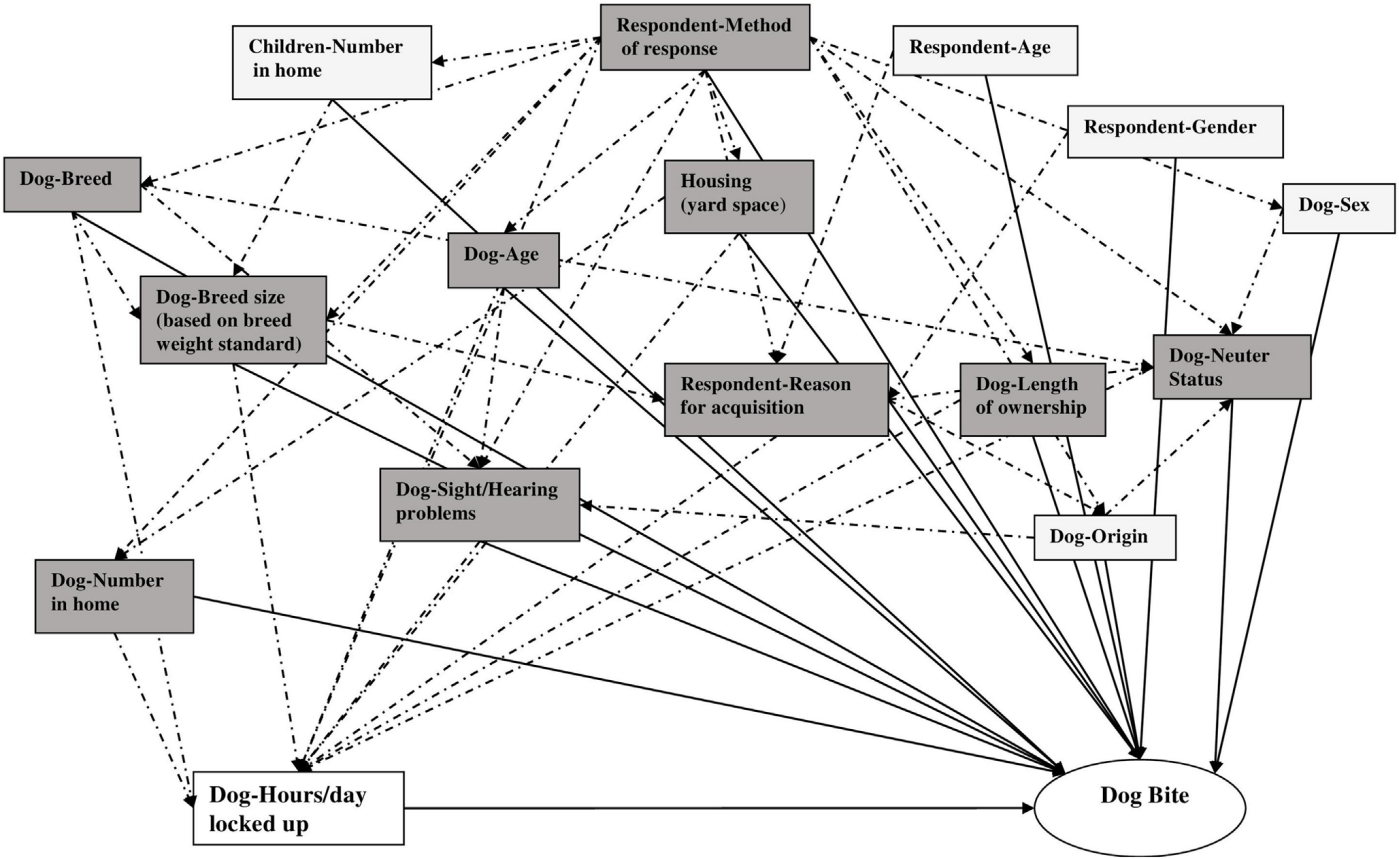
Directed acyclic graph used to select a minimally sufficient set of potential confounders for control of the association of “Number of hours per day locked in kennel, crate or room” with dog bites to the family child. Solid lines represent hypothesized causal relationships between exposures and dog bites. Dotted lines represent hypothesized causal relationships between exposures. All shaded boxes together form a sufficient set of variables for confounder control. All darkly shaded boxes together form a minimally sufficient set of variables for confounder control.

First, a comprehensive directed acyclic graph (DAG) (21) was created incorporating all exposures of interest and potential confounders for which information was available (Figure 1). We then used Dagitty version 2.3 (22) to identify minimally sufficient sets of potential confounders for each exposure of interest (Table 1 and example in Figure 2). In each sufficient set, we included a variable indicating whether or not the respondent had answered alone, as this was thought to be a confounder, i.e., a determinant in identifying a dog bite and also related to the exposures under consideration (23). Log-binomial regression was then employed to estimate the relative risks (RRs) and 95% confidence intervals (CIs) for the association of each exposure of interest with dog bites (24). Using forward selection and the change-in-estimate procedure (25), for each exposure of interest, we selected potential confounders one at a time from its respective DAG-based set (Table 1) for inclusion in the model. For retention in a model, addition of a potential confounder had to result in a change in the RR estimates of at least 10% (26). All continuous variables were added to models as linear terms, as initial analyses using fractional polynomials (27) confirmed that this form produced the best model fit. In estimating the RR of child bites for a given exposure of interest, we excluded all individuals who had missing values for any variables in its DAG-based subset of potential confounders. This was necessary to ensure that changes in RR estimates did not result from changes in numbers of missing observations, as potential confounders were added to or deleted from the model (28). In order to test for differences in exposure-dog bite associations attributable to city of origin, an interaction term consisting of the exposure of interest and city of origin was included in each model. This was retained if the *p*-value was 0.1 or less and the differences in RR between cities were substantial. Where there was no evidence of differences attributable to city of origin, we estimated a pooled RR. In order to test the assumption that risk factors for bites occurring “during play” and bites occurring outside of play were etiologically similar, we re-fit all final models, omitting data from participants bitten outside of play and compared the resulting RRs to those from the models based on both types of bites. The RRs from both models were similar in magnitude and direction and the limits of each 95% CI obtained from a model based on both types of bites were completely nested within the corresponding model based solely on just bites occurring “during play.” We, therefore, used the models with both types of bites for inferences.

**Table 1:** Variables included in each hypothesized minimally sufficient set of confounders during the regression procedure analyzing risk factors for bites to a child from the family dog.

Exposures	Hypothesized sufficient set of potential confounders
*By characteristics of the child and child–dog interactions*	
Child’s gender	r3
Physical or mental disability	c1, r3
Major reason for getting dog	d7, r1, r2, r3
Dog avoids child?	c1, c3, c4, c5, c6, c7, d1, d3, d4, d5, d6, d7, d8, e1, e3, e4, e6, e7, r3
*By characteristics of the dog*	
Dog’s origin	r3, r4
Dog’s sex and neuter status	r3
Breed	r3
*By characteristics of the child–dog home environment*	
Number of dogs in home	e3, r3
Housing	
Dog in house?	d2, d3, d4, d5, d6, d7, d8, e2, e3, e6, r3, r4
Dog sleeps in family member’s bedroom?	d3, d4, d6, d7, d8, r3, r4
Dog chained?	d2, d4, d5, d6, d7, d8, e2, e3, r3, r4
Dog locked in kennel, pen, crate, or room?	d2, d4, d5, d6, d7, d8, e2, e3, r3, r4
Dog can leave premises unaccompanied?	d2, d4, d5, d6, d8, e2, e3, e4, e6, e7, r3, r4

*r1-respondents age; r2-respondents gender; r3-method of response; r4-reason for dog acquisition; c1-child’s gender; c3-physical/mental disability?; c4-frequency of energetic play with dog; c5-frequency of petting dog?; c6-touch dog’s food while eating; c7-touch dog while asleep; d1-dog’s origin; d2-dog’s sex/neuter status; d3-dog’s age at acquisition; d4-dog’s current age; d5-length of ownership; d6-dog breed; d7-dog size; d8-dog sight/hearing problems; d9-dog sleeps in family member’s bedroom; e2-number of dogs; e3-housing; e4-dog in house?; e6-dog chained?; e7-dog locked up?; e8-number of dogs in home; e9-dog avoids child?; e10-dog can leave premises unaccompanied*.

We used a magnitude-based approach to inferences as suggested by Braitman (29) and Batterham and Hopkins (30). We selected thresholds of the RR, which we felt would be of practical importance in the context of dog bites to children in the home. RRs of magnitudes consistent with a 25% or more increase in dog-bite incidence (RR ≥ 1.25) and less than 0.8 (the inverse of 1.25) were considered of practical importance. Thus, we used the following classifications:

(a) RR ≥ 1.25—substantial positive association (of practical importance)
(b) 0.80 < RR < 1.25—weak association (of no practical importance)
(c) RR ≤ 0.80—substantial negative association (of practical importance)

While RRs ≥ 1.25 or ≤0.80 might not be considered practically important in every context, we based our categorizations on the following reasoning:

1. The victim of the bite is a vulnerable individual, a minor.
2. The injury occurs in the child’s home, where the child should be safe from harm.
3. The perpetrator of the injury, the dog, is a member of the child’s household.
4. The consequences of the injury negatively affect the welfare of the dog, in addition to the wellbeing of the victim.

**Figure 3:**
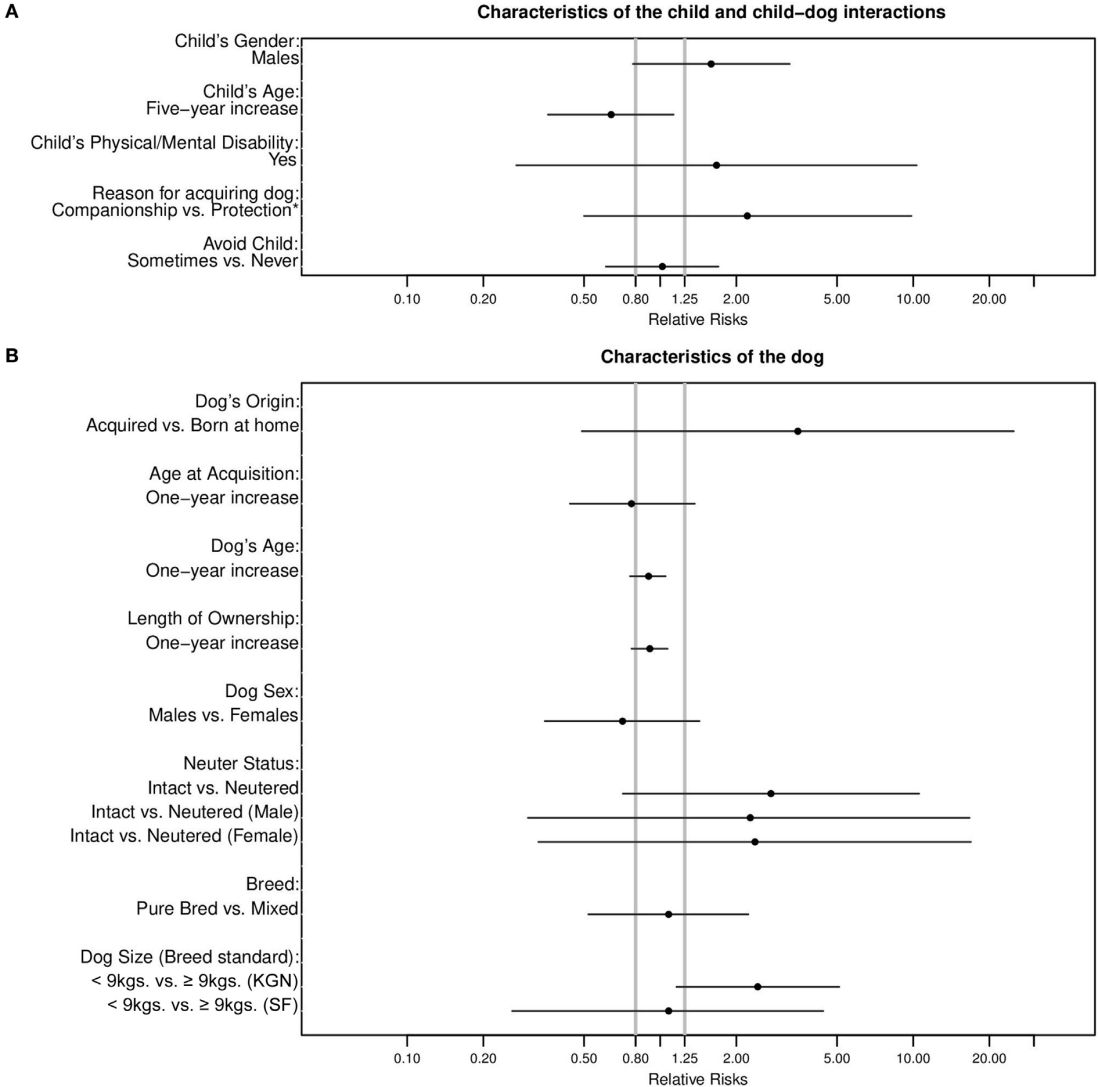

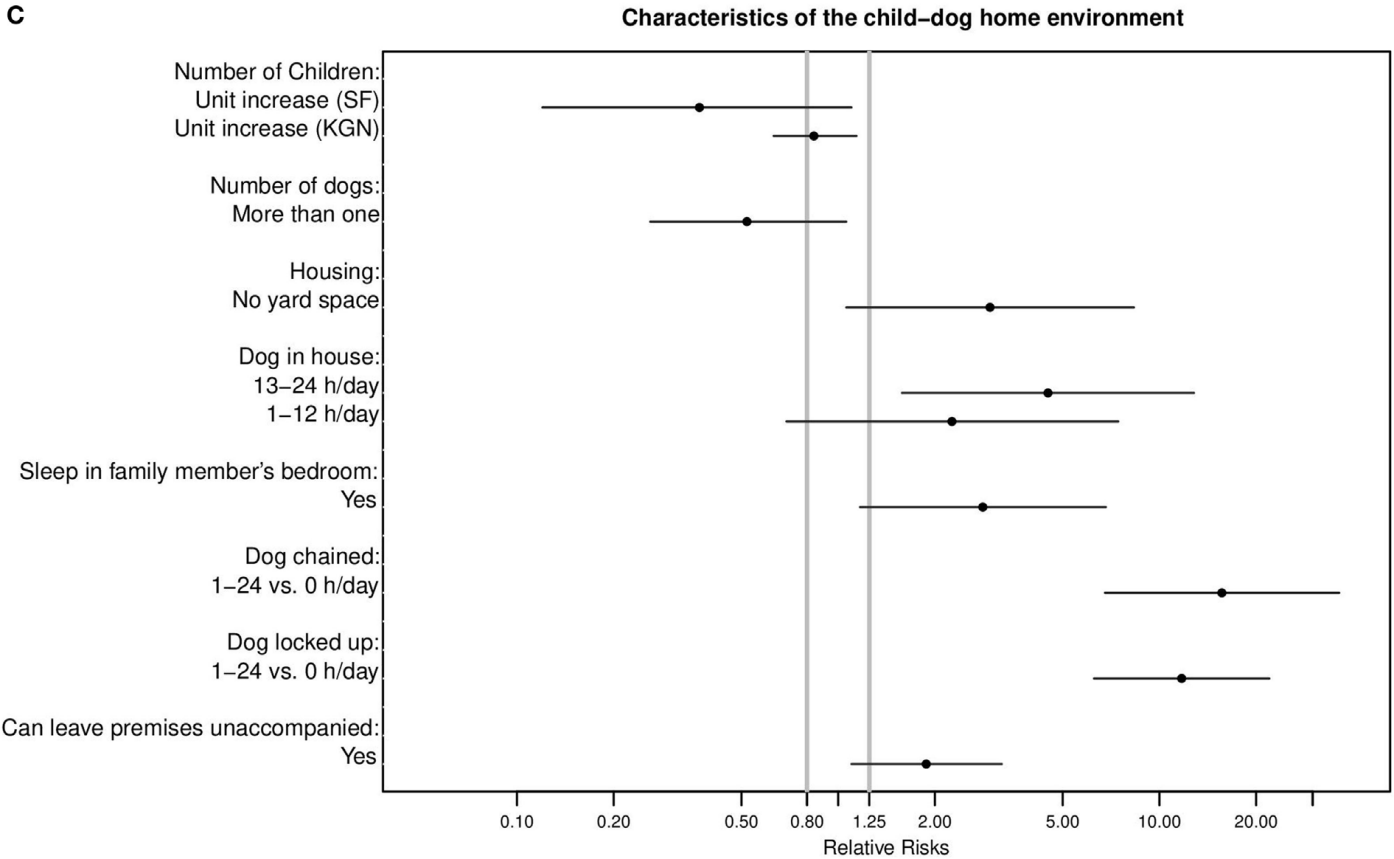
Location of relative risk estimates and associated 95% confidence intervals for bites to a child by a dog living in the same home with respect to threshold values of RR = 0.8 and 1.25 by **(A)** characteristics of the child and child–dog interactions, **(B)** characteristics of the dog, and **(C)** characteristics of the child−dog home environment.

To derive our inferences:

(a) First, we compared the magnitude of the estimated RRs, and the location and width of each 95% CI to the RR threshold (Figure 3). Specifically, we qualitatively evaluated the extent to which each 95% CI contained RR values, which were or were not consistent with RRs of practical importance.
(b) Second, we used the results of our frequentist analysis to estimate the probability (Prob) that, based on our data and vague prior information on the magnitude of the exposure–bite relationships, the population RRs were at least 1.25 [Prob(RR ≥ 1.25)] or no greater than 0.80 [Prob(RR ≤ 0.80)], for positive and negative associations, respectively. To estimate these probabilities, we used a MS Excel spreadsheet [Available at: http://www.sportsci.org/resource/stats/xcl.xls (“3. Rate Ratio and other Log-Normally Distributed Effect Statistics”)] (31). The spreadsheet makes use of the result that for a given likelihood function a conventional X% confidence interval corresponds directly to a Bayesian X% probability interval when the Bayesian analysis is conducted using a uniform prior distribution (32–34). This direct congruence legitimizes the use of confidence intervals to generate probabilistic statements under assumptions of vague prior knowledge (32, 33, 35).
(c) Third, for each exposure–dog bite relationship, we qualitatively described the probability of the population RR exceeding the specified value, applying a modification of the scheme (Table 2) proposed by Hopkins (36). Thus for example, if Prob(RR ≥ 1.25) = 78%, the positive association was deemed “likely of practical importance” and if Prob(0.80 < RR < 1.25) = 97%, the association was deemed very likely of no practical importance (Table 2).

**Table 2:** Qualitative interpretations of the probabilities that the population RR lies in the given ranges.

Probability (%)	Practically important RR ≥ 1.25 or ≤ 0.8	Not practically important 0.80 < RR < 1.25
≤1	Almost certainly not	
>1–25	Very unlikely	
>25–50	Unlikely	
>50–75	Possibly	
>75–95	Likely	
>95	Very likely	

*Adapted with modification from Hopkins, 2002 (36)*.

## Results

### Demographic Information

Data for 236 (79%) Kingstonian and 61 San Franciscan (21%) child–dog pairs were analyzed. Over the 2-year period, the incidence of bite events was 9 and 11 per 100 dog–child pairs in KGN and SF, respectively. Demographic information for both the respondents and participants is displayed in Table 3. Slightly more than half of the respondents were females, with approximately equal distributions in both cities (KGN: 55%, SF: 57%). San Franciscan respondents were older than Kingstonian respondents (67% >40 years vs. 42% >40 years) and slightly fewer answered with the help of another person (34 vs. 38%, respectively). Almost all Kingstonian (97%) and San Franciscan (90%) respondents answered by themselves or jointly with another member of their household (Table 3). Among respondents reporting a bite, this percentage was 100% in both jurisdictions (Table 3). Homes in KGN tended to have more children below the age of 18 years than those in SF, with median (M) and inter-quartile ranges (Q_1_–Q_3_) of *M* = 2; Q_1_–Q_3_ = 1–3 and *M* = 2; Q_1_–Q_3_ = 1–2, respectively. Kingstonian child participants were older (*M* = 10.9 years; Q_1_–Q_3_ = 7.9–12.8 years) than their SF counterparts (*M* = 9.5 years; Q_1_–Q_3_ = 7.4–13.0 years). KGN homes also had more dogs than SF homes (*M* = 2 dogs; Q_1_–Q_3_ = 1–4 dogs vs. *M* = 1 dog; Q_1_–Q_3_ = 1–2 dogs). Compared to those in SF, dogs in KGN homes generally were acquired earlier (93 vs. 78% ≤6 months), were younger (59% vs. 37% ≤6 months), and had been owned for slightly less time (35 vs. 34% ≤2 months). Additionally, fewer Kingstonian (46%; 95% CI: 39–52%) compared to San Franciscan (70%; 95% CI: 59–82%) dogs were acquired for reasons that included companionship but not protection.

**Table 3:** Distribution of biting and non-biting dogs by exposure status and city of origin: Kingston (KGN), Jamaica, and San Francisco (SF), USA.

Exposure	Exposure categories	Bites		Non-bites	
		KGN *n*(%)^a^	SF *n*(%)^a^	KGN *n*(%)^a^	SF *n*(%)^a^
*By characteristics of the respondents*					
Respondent’s age (years)	≤20	1 (4)	1 (14)	4 (2)	0 (0)
	21–30	5 (23)	0 (0)	30 (14)	5 (9)
	31–40	10 (45)	3 (43)	85 (40)	11 (21)
	41–50	4 (18)	2 (29)	61 (29)	28 (53)
	51–60	2 (9)	1 (14)	18 (8)	7 (13)
	61–70	0 (0)	0 (0)	12 (6)	1 (2)
	≥71	0 (0)	0 (0)	2 (1)	1 (2)
	**Total: 294**	**22**	**7**	**212**	**53**
Respondent’s gender	Male	6 (27)	2 (29)	101 (47)	25 (46)
	Female	16 (73)	5 (71)	113 (53)	29 (54)
	**Total: 297**	**22**	**7**	**214**	**54**
Method of response	Alone	13 (59)	2 (29)	133 (62)	38 (70)
	Spouse/companion helped	2 (9)	0 (0)	18 (8)	4 (7)
	Child helped	7 (32)	5 (71)	55 (26)	9 (17)
	Other individual helped	0 (0)	0 (0)	8 (3)	3 (6)
	**Total: 297**	**22**	**7**	**214**	**54**
Major reason for getting dog	Included protection (no comp.)^b^	2 (9)	0 (0)	48 (22)	0 (0)
	Included comp.(no protection)^c^	14 (64)	5 (71)	94 (44)	38 (70)
	All other combinations	6 (27)	2 (29)	72 (34)	16 (30)
	**Total: 297**	**22**	**7**	**214**	**54**
*By characteristics of the child and child–dog interactions*					
Child’s gender	Male	14 (64)	3 (43)	103 (48)	25 (46)
	Female	8 (36)	4 (57)	110 (52)	29 (54)
	**Total: 296**	**22**	**7**	**213**	**54**
Physical or mental disability	Yes	1 (4)	0 (0)	1 (<1)	4 (8)
	No	21 (96)	7 (100)	206 (>99)	47 (92)
	**Total: 297**	**22**	**7**	**214**	**54**
*By characteristics of the dog*					
Dog’s origin	Born at home	1 (4)	0 (0)	33 (15)	0 (0)
	Acquired	21 (96)	7 (100)	181 (85)	54 (100)
	**Total: 297**	**22**	**7**	**214**	**54**
Dog’s sex and neuter status	Male (intact)	7 (32)	4 (57)	98 (46)	14 (26)
	Male (castrated)	1 (4)	0 (0)	5 (2)	19 (36)
	Female (intact)	14 (64)	2 (29)	105 (49)	7 (13)
	Female (spayed)	0 (0)	1 (14)	5 (2)	13 (24)
	**Total: 295**	**22**	**7**	**213**	**53**
Breed	Pure bred	5 (23)	5 (71)	61 (29)	36 (67)
	Mixed	17 (77)	2 (29)	152 (71)	18 (33)
	**Total: 296**	**22**	**7**	**213**	**54**
Dog breed size (based on breed standard)^d^	≥9.0 kg (20 lbs)	7 (32)	4 (57)	106 (49)	32 (59)
	<9.0 kg (20 lbs)	11 (50)	3 (43)	42 (20)	22 (41)
	Unknown	4 (18)	0 (0)	66 (31)	0 (0)
	**Total: 297**	**22**	**7**	**214**	**54**
Sight/hearing problems	Yes	0 (0)	0 (0)	6 (3)	6 (12)
	No	22 (100)	7 (100)	205 (97)	44 (88)
	**Total: 290**	**22**td	**7**	**211**	**50**
Avoid child	≥50% of the time	1 (5)	0 (0)	5 (2)	3 (6)
	<50% of the time	2 (9)	2 (29)	22 (11)	9 (18)
	Never	19 (86)	5 (71)	182 (87)	38 (76)
	**Total: 288**	**22**	**7**	**209**	**50**
*By characteristics of the child–dog home environment*					
Number of dogs	1 dog	11 (50)	5 (71)	62 (30)	36 (68)
	>1 dog	11 (50)	2 (29)	148 (70)	17 (32)
	**Total: 292**	**22**	**7**	**210**	**53**
Housing	Yard space	21 (95)	5 (71)	211 (99)	47 (89)
	No yard space	1 (5)	2 (29)	2 (1)	6 (11)
	**Total: 295**	**22**	**7**	**213**	**53**
Dog in house (h/day)	19–24	10 (45)	7 (100)	42 (20)	29 (55)
	13–18	1 (5)	0 (0)	10 (5)	12 (23)
	7–12	0 (0)	0 (0)	12 (6)	6 (11)
	1–6	7 (32)	0 (0)	51 (24)	4 (7)
	0	4 (18)	0 (0)	99 (46)	2 (4)
	**Total: 296**	**22**	**7**	**214**	**53**
Dog sleeps in family member’s bedroom?	Yes	8 (36)	6 (86)	26 (12)	27 (51)
	No	14 (64)	1 (14)	188 (88)	26 (49)
	**Total: 296**	**22**	**7**	**214**	**53**
Dog chained? (h/day)	19–24	0 (0)	0 (0)	6 (4)	0 (0)
	13–18	3 (14)	0 (0)	2 (1)	0 (0)
	7–12	0 (0)	0 (0)	12 (4)	0 (0)
	1–6	17 (77)	1 (14)	11 (3)	1 (2)
	0	2 (9)	6 (86)	183 (88)	52 (98)
	**Total: 296**	**22**	**7**	**214**	**53**
Dog locked up? (h/day)	19–24	1 (4)	1 (14)	24 (11)	0 (0)
	13–18	1 (4)	0 (0)	7 (3)	2 (4)
	7–12	3 (14)	2 (29)	30 (14)	11 (21)
	1–6	15 (68)	0 (0)	7 (3)	4 (7)
	0	2 (9)	4 (57)	146 (68)	36 (68)
	**Total: 296**	**22**	**7**	**214**	**53**
Dog can leave premises unaccompanied?	Yes	9 (41)	1 (14)	34 (16)	2 (4)
	No	13 (59)	6 (86)	178 (84)	50 (96)
	**Total: 293**	**22**	**7**	**212**	**52**

*^a^Percentages don’t add to 100 due to rounding error*.

*^b^Included protection and other reasons (e.g. “love dogs,” “to take care of dog,” etc.) but not companionship*.

*^c^Included companionship and other reasons (e.g. “love dogs,” “to take care of dog,” etc.) but not protection*.

*^d^Based on breed standards (37, 38)*.

### Location and Widths of 95% CIs With Respect to the Hypothesized Population RR

RR estimates for most exposure–bite relationships were imprecise, though consistent with population RRs ≥ 1.25 or ≤0.8 (Figure 3).

### Characteristics of the Child and Child–Dog Interactions

Males were 1.59 times more likely (95% CI: 0.78–3.25) to be bitten than females with Prob(RR ≥ 1.25) = 75% (Figure 3A; Table 4). The risk of being bitten was inversely related to the child’s age (RR = 0.64; 95% CI: 0.36–1.13 for a 5-year increase in age) with Prob(RRs ≤ 0.8) = 78%. Dogs that were obtained for companionship and other reasons excepting protection were 2.21 (95% CI: 0.50–9.84) times more likely to bite [Prob(RR ≥ 1.25) = 77%] than dogs that were obtained for protection and other reasons excluding companionship. Dogs that sometimes avoided the child were no more likely to have bitten that child than those that never avoided the child (Figure 3A; Table 4).

**Table 4:** Adjusted relative risks (RRs), 95% confidence intervals (CIs), confounders (C) causing ≥ 10% change in RRs, and probabilities that population RRs [Prob(RR)] lie in the given range, for associations between selected variables and family dog-family child bite incidents, Kingston (KGN), Jamaica, and San Francisco (SF), USA.

Exposure	Exposure categories	RR	95% CI	C	Prob(RR) (%)		
					≤0.8	>0.8 – <1.25	≥1.25
*By characteristics of the child and child–dog interactions*							
Child’s gender	Males	1.59	0.78–3.25		3	22	75
	Females	1					
	**Total: 296^a^**						
Physical or mental disability	Yes	1.67	0.27–10.32		22	16	62
	No	1					
	**Total: 296^a^**						
Major reason for getting dog	Included protection (no comp.)^b^	0.55^d^	0.12–2.57	d7	68	17	15
	Included comp.(no protection)^c^	1.22^d^	0.54–2.78		15	37	48
	All other combinations	1					
	**Total: 296^a^**						
Avoid child	Sometimes	1.02	0.61–1.70	d7, e7	17	61	22
	Never	1					
	**Total: 214^a^**						
*By characteristics of the dog*							
Dog’s origin	Acquired	3.5^d^	0.49–24.98		7	8	85
	Born at home	1					
	**Total: 296^a^**						
Dog’s sex and neuter status	Male (intact)	1.71	0.23–12.52		23	15	62
	Male (castrated)	0.76	0.05–11.38		51	13	36
	Female (intact)	2.37	0.33–16.89		14	12	74
	Female (spayed)	1					
	**Total: 296^a^**						
Breed	Pure bred	1.08	0.52–2.23		21	44	35
	Mixed	1					
	**Total: 295^a^**						
*By characteristics of the child–dog home environment*							
Number of dogs in home	More than one	0.52	0.26–1.06		88	11	1
	One	1					
	**Total: 291^a^**						
Housing	No yard space	2.97	1.06–8.33		1	4	95
	Yard space	1					
	**Total: 294^a^**						
Dog in house? (h/day)	13–24	4.5	1.58–12.81	d2, d7	<0.1	1	99
	1–12	2.26	0.69–7.45		4	12	84
	0	1					
	**Total: 272^a^**						
Sleep in family member’s bedroom?	Yes	2.82	1.17–6.81	d4, d7	<0.5	3	97
	No	1					
	**Total: 270^a^**						
Dog chained? (h/day)	1–24	15.65^b^	6.77–36.28	e3	0	0	>99.9
	0	1					
	**Total: 266^a^**						
Dog locked in kennel, pen, crate, or room? (h/day)	1–24	11.73	6.26–21.99	e3	0	0	>99.9
	0	1					
	**Total: 266^a^**						
Can leave premises	Yes	1.88	1.10–3.23	e7	0.1	6.8	93.1
Unaccompanied?	No						
	**Total: 264**^a^	1					

*^a^Total number of participants (297) less the number with missing data for at least one of the variables in the necessary set of confounders*.

*^b^Included protection and other reasons (e.g., “love dogs,” “to take care of dog,” etc.) but not companionship*.

*^c^Included companionship and other reasons (e.g., “love dogs,” “to take care of dog,” etc.) but not protection*.

*^d^RR heavily influenced by Kingston data*.

d2, dog’s sex/neuter status; d4, dog’s current age; d7, dog size; e3, housing; e7, dog locked up?

### Characteristics of the Dog

The age of the dog at acquisition was inversely related to a child being bitten (RR = 0.77: 95% CI: 0.44–1.37—for a 1-year increase). Conversely, dogs that were acquired (as opposed to being born in their current owner’s home) were at higher risk (RR = 3.5: 95% CI: 0.49–24.98) for biting than dogs that were not (Figure 3B; Table 4). Both 1-year increases in dog age (RR = 0.90: 95% CI: 0.76–1.05) and length of ownership (RR = 0.91: 95% CI: 0.77–1.07) showed inverse associations with bites. Intact dogs were at overall higher risk for biting (RR = 2.74; 95% CI: 0.71–10.55) than neutered [Figure 3B and Prob(RR ≥ 1.25) = 87%]. This was also true when males (RR = 2.25; 95% CI: 0.3–16.67) and females (RR = 2.37; 95% CI: 0.30–16.89) were considered separately (Figure 3B; Table 4) with Prob(RR ≥ 1.25) = 72 and 74%, respectively. In KGN, smaller breeds (<9 kg or 20 pounds) were at higher risk for biting (RR = 2.43; 95% CI: 1.16–5.10) than larger breeds (≥9 kg or 20 pounds), but not so in SF (RR = 1.08; 95% CI: 0.26–4.41) (Figure 3B). The Prob(RR ≥ 1.25) for the KGN and SF comparisons were 96 and 42%, respectively. No dog with a sight or hearing problem had bitten a child in the preceding 2 years (Table 1).

### Characteristics of the Child–Dog Home Environment

The risk of a child bite was inversely associated with the number of children in the home, though more so in SF (RR = 0.37; 95% CI: 0.12–1.10) than in KGN (RR = 0.84; 95% CI: 0.63–1.14) (Figure 3C). The Prob(RR ≤ 0.80) and Prob(0.80 < RR < 1.25) for the SF and KGN comparisons were 91 and 62%, respectively. Similarly, bites were inversely associated with the number of dogs present in the home (Figure 3C; Table 4). Dogs that lived in a home with no yard space were at elevated risk of biting (RR = 2.97; 95% CI: 1.06–8.33) compared to dogs that had yard space (Figure 3C). Dogs allowed inside for some portion of the day (1–24 h) were three times as likely to bite a child living in the home (95% CI: 0.94–9.62) than those that were not [Prob(RR ≥ 1.25) = 93%]. Additionally, dogs that spent 13–24 h a day inside were approximately twice as likely to bite as those that spent 1–12 h per day (Table 4). Both these groups were at higher risk for biting than those that were not allowed inside (Table 4). Both chaining and confining to a kennel, pen, crate, or room for some portion of the day showed strong associations with child bites [Prob(RR ≥ 1.25) > 99.9%] though the 95% CIs were wide (Table 4; Figure 3C). Finally, a dog being able to leave the premises unaccompanied was positively associated with biting a child in the home (Figure 3C; Table 4).

## Discussion

Studies of risk factors for dog bites are generally either dog- (16–18, 39–41) or victim-focused (42–44). This study differs from most others in placing equal emphasis on victim (child)- and dog-related factors contributing to a child bite. Additionally, as this study population is nested within the study population of a larger cohort study on dog bites, it facilitates comparisons of these results to previous findings on risk factors for general bites (16–18).

The associations with bites to children found for “lack of yard space,” “increased hours spent by the dog inside,” and “routinely sleeping in a family member’s bedroom” are likely substantial and of practical importance [Prob(RR ≥ 1.25) ≥ 90%]. These associations are similar to those found for bites in general (Table 5). A history of sleeping in a family member’s bed has also previously been found to be associated with bites to owners (41). It is probable that these effects are mediated through the frequency of child–dog interaction. If so, it seems paradoxical that increased chaining or confinement are also positively associated with relative risks for biting the child that are very likely of practical importance [Prob(RR ≥ 1.25) > 99.9%] (Figure 3C; Table 4). One possible explanation is that while chaining and confinement might effectively restrict the interaction of dogs with non-household members, the same is not necessarily true for its interaction with a child that lives in the home. In fact, if not properly monitored, chaining and confinement may just limit the dog’s ability to retreat from the child if it wishes to, and thus increase the risk of a bite incident. This could potentially explain the increased RRs compared to the general cohort (16) (Table 5). It is also possible that some dogs might be routinely chained or confined because they may have bitten the child previously. If so, this raises the possibility of temporal bias (45). Comprehensively, while we do not know whether these bites actually occurred within the context of such events (i.e., while being inside the house, sleeping in a family member’s bedroom, while being chained or confined etc.), these results may indicate that these management factors are positively correlated with other factors that result in dog bites.

**Table 5:** Adjusted relative risks (RR) and 95% confidence intervals for associations between selected variables and dog bites in general, Kingston (KGN), Jamaica, and San Francisco (SF) (Adapted from Messam et al., 2008) (16).

Exposure (sample size)	Exposure categories	RR	95% CI
*By characteristics of the child and child–dog interactions*			
Major reason for getting dog (1100)	Included protection (no comp.)^a^	0.82^c^	0.49–1.38
	Included comp. (no protection)^b^	1.36^c^	0.99–1.99
	All other combinations	1	
*By characteristics of the dog*			
Dog’s origin (1100)	Acquired	1.41	0.8–2.44
	Born at home	1	
Dog’s sex and neuter status (1026)	Male (intact)	2.56	1.51–4.34
	Male (castrated)	1.52	0.94–2.46
	Female (intact)	3.22	1.86–5.59
	Female (spayed)	1	
*By characteristics of the child–dog home environment*			
Housing (1101)	No yard space	1.16^d^	0.77–1.75
	Yard space	1	
Dog in house (h/day) (1044)	19–24	1.97^c^	1.17–3.32
	13–18	1.90^c^	0.99–3.62
	7–12	2.18^c^	1.18–4.02
	1–6	1.00^c^	0.51–1.96
	0	1	
Sleep in family member’s bedroom (1042)	Yes (KGN)	2.54	1.43–4.54
	Yes (SF)	1.11	0.67–1.85
	No	1	
Dog chained/leashed (h/day) (974)	1–24	1.15	0.66–1.99
	0	1	
Dog locked in kennel, pen, crate, or room (h/day) (973)	19–24	0.44	0.07–2.76
	13–18	0.93	0.35–2.46
	7–12	1.15	0.72–1.83
	1–6	1.71	1.02–2.86
	0	1	
Can leave premises unaccompanied (1042)	Yes (KGN)	1.04	0.63–1.72
	Yes (SF)	3.40	1.98–5.85
	No	1	

*^a^Acquired for protection or for protection and other reasons excluding companionship*.

*^b^Acquired for companionship or for companionship and other reasons excluding protection*.

*^c^RR heavily influenced by KGN data*.

*^d^RR heavily influenced by SF data*.

The finding that male children are more likely to be bitten than females is consistent with previous reports (4, 5, 44). It has been suggested that gender-based differences in the nature of human–dog interactions play an etiological role in differences in dog-bite frequency between males and females (15). If true, this is likely to be relevant in the home environment as well. These results suggest that this association is possibly of practical importance. The observed inverse relationship between child-bite risk and child-age is likely due to a combination of increased size, increased knowledge of dogs, and less unpredictable behavior on the part of the child (4, 15). The true (population) effect of 5-year increases in child age is a likely substantial reduction in dog bite risk [Prob(RRs ≤ 0.8) = 78%]. Dogs obtained for reasons that included companionship but not protection are likely at substantially higher risk for biting a child [Prob(RR > 1.25) = 77%] even after controlling for breed size. This is consistent with the results for general bites in the larger cohort as evident from the similarity of the corresponding RR estimates and overlap in the 95% CIs (Tables 4 and 5) (16). While these results might still be explained, in part, by residual confounding by breed, parents may also be more watchful and/or restrictive of children’s interactions with a dog obtained for household protection. Data from Kingstonian participants disproportionately influenced these results as no SF dogs were obtained for reasons that included protection but not companionship (Table 3).

The inverse, though likely weak association [Prob(0.80 < RR < 1.25) = 93%] between dog age and bites to the family child is consistent with estimates from other studies (41, 43) but different to our findings in the larger cohort (17). It is reasonable to expect a substantial positive association between dog age and dog bites because of the relationship between age and the development of canine aggressive behavior. As we have mentioned elsewhere (17), a weak observed dog age–dog bite association could be attributable to age being used in the analysis in linear, as opposed to in polynomial form, as was used in the analysis of the larger cohort’s data (17). Similar results for length of ownership (essentially the time the dog has lived in the home environment) can be explained by its high correlation (Pearson’s correlation coefficient = 0.91; 95% CI: 0.84–0.98) with dog age. Higher risks for general bites observed for intact, compared to neutered dogs have been previously observed in the larger cohort (Table 5) (16) and by other authors (39, 41). This study’s results suggest that the association between bites and neuter status is likely substantial and of practical importance [Prob(RR ≥ 1.25) = 87*%*]. The finding that acquired dogs were likely at substantially higher risks for bites [Prob(RR ≥ 1.25) = 90%] than dogs born into their current owner’s home is also consistent with findings in the larger cohort (Table 5). Lower risks for dogs born into their current owner’s home could plausibly result in part from the positive socializing effects of spending a longer time in the maternal environment and/or not experiencing the trauma of changing home (46). A recent review, highlighting increased risks for biting by intact compared to neutered dogs has suggested that, in addition to education, mandatory neutering of dogs might reduce dog bite frequency (47). This would preclude the realization of any beneficial effects on dog bite frequency by dogs being born into their owner’s home in those jurisdictions in which it is currently practiced. Additionally, based on recent data from the United States, early neutering could have adverse effects on dog health especially for some large breeds (48, 49).

It is not clear why smaller breeds in KGN were likely at substantially higher risk for biting but not in SF or why the association between the number of children in the home and bites was likely substantial in SF but not KGN. However, these results suggest that there may be local conditions acting to modify these relationships. Consistent with our findings in KGN, a study in Canada found that smaller dogs were more likely to bite family members than larger dogs (41). A contributing factor may be that smaller breeds in general tend to be more reactive with a higher activity level than larger breeds (37). The Canadian researchers also found that bites were positively associated with the number of teenagers in the home (41), contrary to our findings in both KGN and SF.

### Limitations

This study has a number of limitations. Small numbers of dog bite cases resulted in low precision of our estimates for most exposures. In addition, this low number of outcomes as well as the low prevalence of some exposures may have mitigated against us detecting other differences in city-specific RRs. Second, we did not have information on some potential important confounders (for example, extent of training of dogs), which might have affected some of our estimates (e.g., time spent in house, chaining, and confinement). Third, our inferences are based on an assumption that RRs ≥ 1.25 and RRs ≤ 0.80 are indicative of substantial population associations. Different thresholds for associations of practical importance could plausibly be used. Nevertheless, we believe that this approach is helpful to the investigation of dog bites. In focusing inferences on the magnitude of the parameter of interest (the RR), we encourage readers to ask and decide for themselves whether or not the observed effects are of practical importance. In addition to being data-based, the probabilistic statements made are based on assumptions of having little prior knowledge of the actual magnitude of the associations of these exposures with family dog bites to the family child. This is commensurate with information currently available on the topic.

## Conclusion

Notwithstanding limitations, this study suggests that the risk of a bite to the family child by the family’s dog is associated with home-environment characteristics. These include factors characteristic of the child, the dog, and the child–dog environment. The study also suggests that the relationships with dog bites, for most exposures examined, were of practical importance and are consistent with population RRs of at least 1.25 and no greater than 0.8, for positive and negative associations, respectively. Finally, these results suggest overlap between risk factors for dog bites to children at home and risk factors for dog bites to the general population.

## Ethics Statement

This study was authorized by the University of California Davis’ Human Subjects Institutional Review Board and respondents provided verbal informed consent.

## Author Contributions

LM conceived the study, collected and analyzed the data, and wrote the initial draft of manuscript. LM, PK, BC, and LH contributed to the design of the questionnaire, the design of the study, and reviewed successive drafts of the manuscript for intellectual content.

## Conflict of Interest Statement

The authors declare that the research was conducted in the absence of any commercial or financial relationships that could be construed as a potential conflict of interest.
